# Supplementary figures and images for: ARPEGGIO: Automated Reproducible Polyploid EpiGenetic GuIdance workflOw
Source: BMC Genomics. 2021 Jul 17;22:547. doi: 10.1186/s12864-021-07845-2 (PMC8285871; doi:10.1186/s12864-021-07845-2)

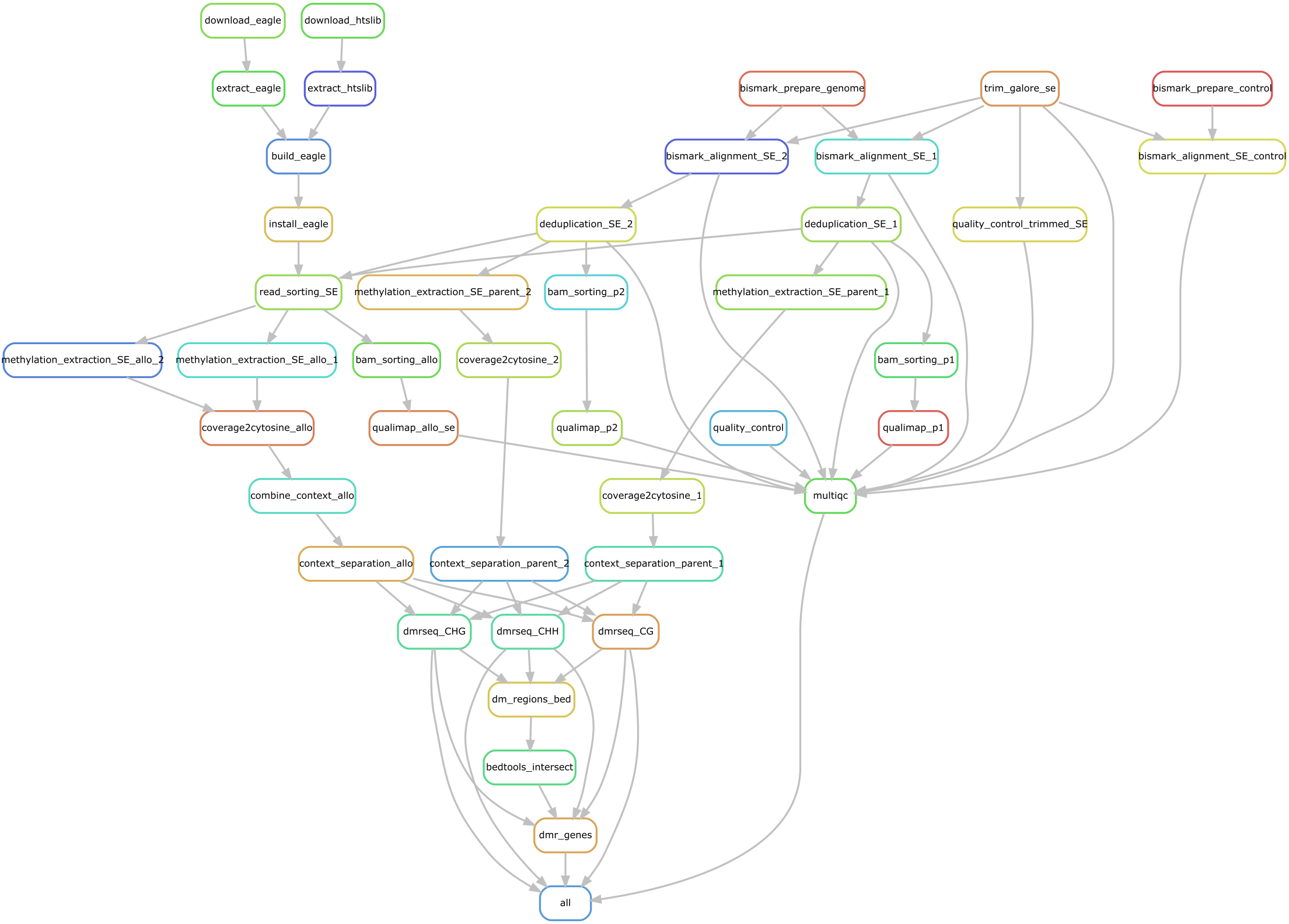

Supplement: Supplementary file 1 — Additional file 1. Example of relationships between rules in ARPEGGIO. Description: A graph showing the input/output relationships between different rules in ARPEGGIO in an example “default” run with single end reads. [file 12864_2021_7845_MOESM1_ESM.pdf]

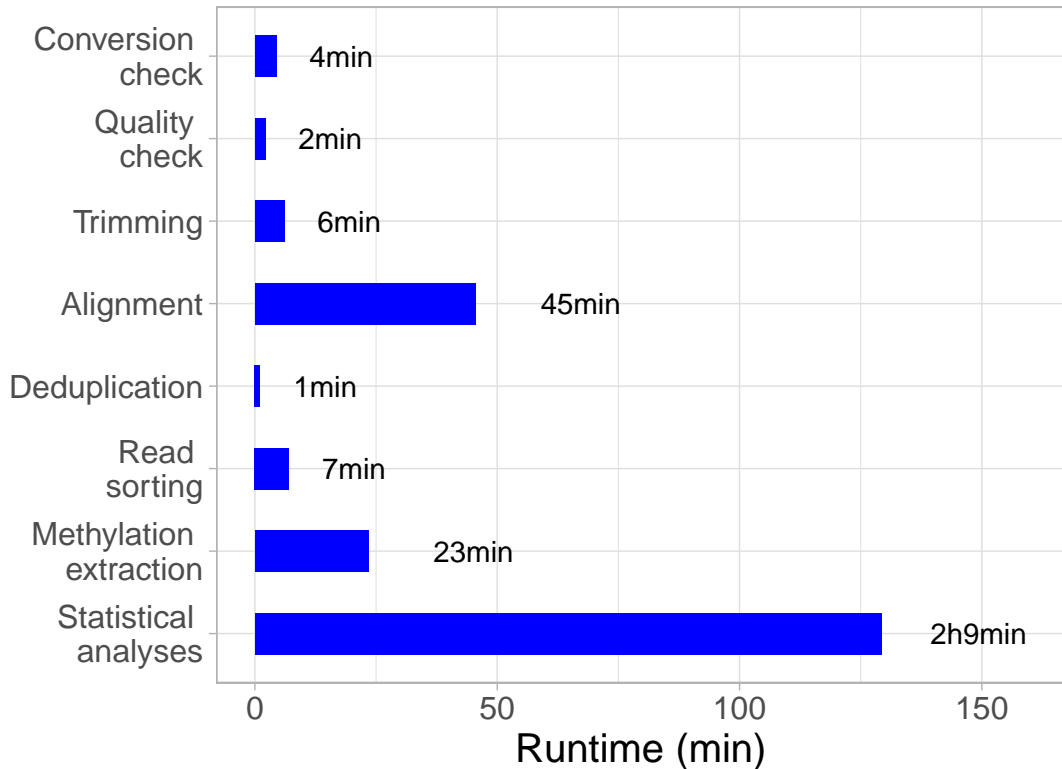

Supplement: Supplementary file 2 — Additional file 2. Plot with average runtimes in ARPEGGIO with Mimulus data. Description: A plot with the average runtime for each main step in the ARPEGGIO pipeline: conversion check, quality check, trimming, alignment, deduplication, read classification, methylation extraction and statistical analyses. Each step shows a per sample average (12 samples in total), with the exception of the statistical analyses step where the average is per methylation context (3 contexts in total). [file 12864_2021_7845_MOESM2_ESM.pdf]
