# Supplementary material for: ARPEGGIO: Automated Reproducible Polyploid EpiGenetic GuIdance workflOw
Source: BMC Genomics. 2021 Jul 17;22:547. doi: 10.1186/s12864-021-07845-2 (PMC8285871; doi:10.1186/s12864-021-07845-2)
Supplement: Supplementary file 3 — Additional file 3. Plant material and WGBS library synthesis. Description: Details about the plant conditions, sampling, DNA extraction, bisulfite treatment and sequencing strategies. [file 12864_2021_7845_MOESM3_ESM.zip › Additional_file_3.pdf]

Plant material and WGBS library synthesis

All plant individuals were incubated in a climate chamber set at 22˚C , with 60% relative humidity, 16 hours light/8hours dark cycles. Mature leaves were collected from each individual, and DNA samples were extracted by DNeasy Plant Mini Kit (Qiagen) from 3 individuals of each species. For the samples *A. halleri* 1 and *A. lyrata* 1, DNA was first treated by bisulfite (BS) using MethylEdge™ Bisulfite Conversion System (Promega), and sequencing library was synthesized by using TruSeq DNA Methylation Kit (Illumina). For the samples *A. halleri* G1 and *A. lyrata* G1, DNA was first synthesized to sequencing library using KAPA HyperPrep Kit with TruSeq DNA Single Indexes Set (Illumina), and BS-treated by EZ DNA Methylation-Gold Kit (ZYMO Research).
The libraries were paired-end sequenced by Illumina HiSeq 4000 (126bp x 2, *A. halleri* 1 and *A. lyrata* 1) and NovaSeq 6000 (150bp x 2, *A. halleri* G1 and *A. lyrata* G1).
