# Supplementary material for: ARPEGGIO: Automated Reproducible Polyploid EpiGenetic GuIdance workflOw
Source: BMC Genomics. 2021 Jul 17;22:547. doi: 10.1186/s12864-021-07845-2 (PMC8285871; doi:10.1186/s12864-021-07845-2)
Supplement: Supplementary file 4 — Additional file 4. Read statistics about datasets used to compare EAGLE-RC against concatenation method. Description: All the numbers related to the datasets used to compare EAGLE-RC to the concatenation method: total reads, uniquely mapped reads (and not), duplicated reads, correct, ambiguous and wrongly classified reads and error rate. [file 12864_2021_7845_MOESM4_ESM.pdf]

Read statistics about datasets used to compare EAGLE-RC against concatenation method

The following tables provide all the numbers behind the comparison between EAGLE-RC against the concatenation method. Here's a list with all the samples used and their corresponding accession number:

- *A. halleri*: SAMD00208469
- *A. lyrata*: SAMD00208470
- *A. halleri* G1: SAMD00208471
- *A. lyrata* G1: SAMD00208472
- *M. luteus* 1, 2 , 3 , 4: SRX2618908, SRX2618909, SRX2618910, SRX2618911
- *M. guttatus* 1 , 2 , 3 , 4: SRX2618912, SRX2618913, SRX2618914, SRX2618915
- *G. arboreum* 1 , 2: SRR3219104, SRR3219105
- *G. raimondii* 1, 2: SRR3219088, SRR3219089

	A. halleri	*A. lyrata*	*A. halleri*	*A. lyrata*
Method	concatenated		EAGLE-RC based classification read sorting	
Total reads (TR)	40’160’266	44’895’570	40’160’266	44’895’570
Uniquely mapped (% from TR)	21’012’844 (52.3%)	23’067’388 (51.4%)	22’119’312 (55.3%)	24’433’418 (54.7%)
Not uniquely mapped	19’147’422	21’828’182	18’040’954	20’462’152
Duplicated reads (% from TR)	5’293’580 (13.2%)	7’585’268 (16.9%)	5’338’990 (24.1%)	7’897’614 (32.3%)
Uniquely mapped and deduplicated (UMD)	15’719’264	15’482’120	16’780’322	16’535’804
Correct reads (% from UMD)	15’013’508 (95.5%)	14’418’656 (91.0%)	14’953’178 (89.1%)	14’417’190 (87.2%)
Ambiguous	-	-	1’633’420	1’859’612
Wrong reads	705’756	1’063’464	193’724	259’002
Error %	4.49 %	6.87%	1.30 %	1.57 %

	A. halleri G1	*A. lyrata* G1	*A. halleri* G1	*A. lyrata G1*
Method	concatenated		EAGLE-RC based classification~~read sorting~~	
Total reads (TR)	80’445’492	124’215’824	80’445’492	124’215’824
Uniquely mapped (% from TR)	20’321’978 (25.3%)	31’692’092 (25.5%)	21’468’920 (26.7%)	32’969’572 (26.6%)
Not uniquely mapped	60’123’514	92’523’732	58’976’572	91’246’252
Duplicated reads (% from TR)	1’523’726 (1.9%)	2’765’528 (2.2%)	1’626’582 (7.6%)	2’903’264 (8.8%)
Uniquely mapped and deduplicated (UMD)	18’798’252	28’926’564	19’842’338	30’066’308
Correct reads (% from UMD)	18’147’370 (96.5%)	27’480’258 (95.0%)	18’625’582 (93.9%)	28’269’980 (94.0%)
Ambiguous	-	-	1’016’786	1’399’008
Wrong reads	650’882	1’446’306	199’970	397’320
Error %	3.46 %	5.00%	1.01 %	1.32 %

	Mimulus guttatus 1	*Mimulus luteus* 1	*Mimulus guttatus* 1	*Mimulus luteus* 1
Method	concatenated		EAGLE-RC based classificationread sorting	
Total reads (TR)	11'198'784	14'540'898	11'198'784	14'540'898
Uniquely mapped (% from TR)	2'612'195 (23.3%)	6'312'383 (43.4%)	2'575'518 (23.0%)	5'908'489 (40.6%)
Not uniquely mapped	8'586'589	8'228'515	8'623'266	8'632'409
Duplicated reads (% from TR)	1'151'447 (44.1%)	2'300'413 (36.44%)	1'249'814 (48.5%)	2'027'318 (34.3%)
Uniquely mapped and deduplicated (UMD)	1'460'748	4'011'970	1'325'704	3'881'171
Correct reads (% from UMD)	1'069'658 (73.2%)	3'623'197 (90.3%)	976'317 (73.6%)	3'236'631 (83.4%)
Ambiguous	-	-	250'067	556'666
Wrong reads	391'090	388'773	99'320	87'874
Error %	26.77%	9.69%	7.49%	2.26%

	Mimulus guttatus 2	*Mimulus luteus* 2	*Mimulus guttatus* 2	*Mimulus luteus* 2
Method	concatenated		EAGLE-RC based classification~~read sorting~~	
Total reads (TR)	11’125’236	14’423’855	11’125’236	14’423’855
Uniquely mapped (% from TR)	2’608’418 (23.4%)	6’291’151 (43.6%)	2’571’997 (23.1%)	5’889’892 (40.8%)
Not uniquely mapped	8’516’818	8’132’704	8’553’239	8’533’963
Duplicated reads (% from TR)	1’149’555 (44.1%)	2’297’202 (36.5%)	1’248’440 (48.5%)	2’026’983 (34.4%)
Uniquely mapped and deduplicated (UMD)	1’458’863	3’993’949	1’323’557	3’862’909
Correct reads (% from UMD)	1’067’813 (73.2%)	3’606’521 (90.3%)	974’850 (73.7%)	3’219’702 (83.3%)
Ambiguous	-	-	249’601	555’449
Wrong reads	391’050	387’428	99’106	87’758
Error %	26.81%	9.70%	7.49%	2.27%

	Mimulus guttatus 3	*Mimulus luteus* 3	*Mimulus guttatus* 3	*Mimulus luteus* 3
Method	concatenated		EAGLE-RC based classification~~read sorting~~	
Total reads (TR)	10’955’877	14’136’379	10’955’877	14’136’379
Uniquely mapped (% from TR)	2’494’759 (22.8%)	6’016’962 (42.6%)	2’459’689 (22.5%)	5’629’101 (39.8%)
Not uniquely mapped	8’461’118	8’119’417	8’496’188	8’507’278
Duplicated reads (% from TR)	1’098’495 (44.0%)	2’193’358 (36.5%)	1’192’556 (48.5%)	1’933’785 (34.4%)
Uniquely mapped and deduplicated (UMD)	1’396’264	3’823’604	1’267’133	3’695’316
Correct reads (% from UMD)	1’022’678 (73.2%)	3’446’647 (90.1%)	932’804 (73.6%)	3’083’952 (83.5%)
Ambiguous	-	-	238’974	526’097
Wrong reads	373’586	376’957	95’355	85’267
Error %	26.76%	9.86%	7.53%	2.31%

	Mimulus guttatus 4	*Mimulus luteus* 4	*Mimulus guttatus* 4	*Mimulus luteus* 4
Method	concatenated		EAGLE-RC based classification~~read sorting~~	
Total reads (TR)	10’646’892	13’738’854	10’646’892	13’738’854
Uniquely mapped (% from TR)	2’433’041 (22.9%)	5’866’025 (42.7%)	2’398’688 (22.5%)	5’485’383 (39.9%)
Not uniquely mapped	8’213’851	7’872’829	8’247’915	8’253’471
Duplicated reads (% from TR)	1’068’453 (43.9%)	2’137’718 (36.4%)	1’159’882 (48.4%)	1’882’322 (34.3%)
Uniquely mapped and deduplicated (UMD)	1’364’588	3’728’307	1’238’806	3’603’061
Correct reads (% from UMD)	998’982 (73.2%)	3’357’807 (90.1%)	911’356 (73.6%)	3’003’804 (83.4%)
Ambiguous	-	-	233’955	515’291
Wrong reads	365’606	370’500	93’495	83’966
Error %	26.79%	9.94%	7.55%	2.33%

	Gossypium arboreum 1	*Gossypium raimondii* 1	*Gossypium arboreum* 1	*Gossypium raimondii* 1
Method	concatenated		EAGLE-RC based classification~~read sorting~~	
Total reads (TR)	432’844’852	356’699’260	432’844’852	356’699’260
Uniquely mapped (% from TR)	279’996’748 (64.7%)	273’814’708 (76.8%)	280’386’284 (64.8%)	278’732’236 (78.1%)
Not uniquely mapped	152’848’104	82’884’552	152’458’568	77’967’024
Duplicated reads (% from TR)	18’846’046 (6.7%)	13’284’372 (4.8%)	18’879’306 (6.7%)	13’697’054 (4.9%)
Uniquely mapped and deduplicated (UMD)	261’150’702	260’530’336	261’506’978	265’035’182
Correct reads (% from UMD)	260’132’278 (99.6%)	259’423’814 (99.6%)	260’022’724 (99.4%)	262’945’068 (99.2%)
Ambiguous	-		1’132’490	1’613’104
Wrong reads	1’018’424	1’106’522	351’764	477’010
Error %	0.00390%	0.00425%	0.00134%	0.00179%

	Gossypium arboreum 2	*Gossypium raimondii* 2	*Gossypium arboreum* 2	*Gossypium raimondii* 2
Method	concatenated		EAGLE-RC based classification~~read sorting~~	
Total reads (TR)	414’743’906	299’026’128	414’743’906	299’026’128
Uniquely mapped (% from TR)	264’247’068 (63.7%)	235’500’124	264’620’650 (63.8%)	240’038’096 (80.3%)
Not uniquely mapped	150’496’838	63’526’004	150’123’256	58’988’032
Duplicated reads (% from TR)	17’572’436 (4.2%)	10’850’322 (4.6%)	17’604’224 (4.2%)	11’202’082 (4.7%)
Uniquely mapped and deduplicated (UMD)	246’674’632	224’649’802	247’016’426	228’836’014
Correct reads (% from UMD)	245’718’300 (99.6%)	223’623’540 (99.5%)	245’626’594 (99.4%)	226’937’338 (99.2%)
Ambiguous	-	-	1’059’410	1’452’404
Wrong reads	956’332	1’026’262	330’422	446’272
Error %	0.00389%	0.00457%	0.00134%	0.00195%
